# Channel and Spatial Parallel Attention for ECG‐Based Prediction of Concealed Accessory Pathways and Atrioventricular Nodal Reentry Tachycardia

**DOI:** 10.1002/joa3.70373

**Published:** 2026-05-25

**Authors:** Lei Wang, Hui Yan, Qi Cheng, Feng Pan, Ru‐Xing Wang, Shipeng Dang

**Affiliations:** ^1^ School of Intelligent Engineering Jiangsu Vocational College of Information Technology Wuxi China; ^2^ Key Laboratory of Advanced Process Control for Light Industry (Ministry of Education) Jiangnan University Wuxi China; ^3^ Department of Cardiology, The Affiliated Wuxi People's Hospital of Nanjing Medical University, Wuxi Medical Center Nanjing Medical University Wuxi China

**Keywords:** atrioventricular nodal reentry tachycardia, channel and spatial parallel attention, concealed accessory pathways, ECG prediction

## Abstract

**Background:**

Concealed accessory pathways (CAP) and atrioventricular nodal reentry tachycardia (AVNRT) represent diagnostically challenging forms of paroxysmal supraventricular tachycardia, with conventional sinus rhythm ECGs often failing to reveal characteristic abnormalities.

**Methods:**

We developed CSPANet, a novel deep learning architecture that integrates a Channel and Spatial Parallel Attention (CSPA) module for enhanced ECG feature extraction. The architecture features parallel processing through two specialized attention mechanisms: a channel attention submodule that adaptively weights clinically significant ECG leads using complementary feature pathways, working in concert with a spatial attention submodule that captures essential morphological patterns through synergistic multi‐scale pooling and convolutional feature extraction.

**Results:**

In a comparative study of nine classical CNNs, ResNet50 demonstrated superior performance, achieving the highest sensitivity and specificity and validating the efficacy of residual learning for this task. The proposed CSPANet, integrating our novel channel and spatial parallel attention (CSPA) mechanism, achieved a test set accuracy of 92.6%, sensitivity of 79.0%, specificity of 95.0%, and precision of 79.7%, surpassing all other representative attention mechanisms. Ablation studies confirmed the individual and synergistic contributions of the CSPA and Stem modules, with their combined integration yielding the most significant performance gains, including an 11.7% increase in sensitivity and an 8.8% increase in precision over the baseline ResNet20 model.

**Conclusion:**

CSPANet's ability to differentiate CAP and AVNRT from sinus rhythm ECGs offers a transformative clinical tool, facilitating optimized ablation planning and enhancing procedural safety. By addressing a key diagnostic gap, this approach underscores the potential of deep learning to refine arrhythmia management.

## Graphical abstract

CSPANet, a deep learning model with channel and spatial parallel attention, distinguishes concealed accessory pathways and atrioventricular nodal reentry tachycardia from sinus‐rhythm ECGs with 92.6% accuracy. This novel approach facilitates optimized ablation planning and enhances procedural safety, addressing a key diagnostic gap in arrhythmia management.

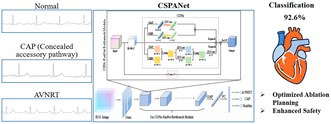

## 1 Introduction

Electrocardiography (ECG) is a fundamental, non‐invasive diagnostic tool widely used for detecting cardiac arrhythmias and conduction disorders [1, 2]. In recent years, deep learning techniques have demonstrated strong potential in analyzing large‐scale ECG data, enabling the identification of subtle morphological changes that are difficult to detect manually, thus supporting early diagnosis and risk stratification of cardiovascular diseases [3, 4].

Atrioventricular nodal reentry tachycardia (AVNRT) and concealed accessory pathways (CAP) are two common subtypes of paroxysmal supraventricular tachycardia (PSVT), with an estimated incidence of 35 per 100 000 individuals annually [5, 6]. These arrhythmias are characterized by sudden‐onset palpitations, tachycardia, dizziness, chest discomfort and, in severe cases, syncope [7, 8]. Their episodic and transient nature significantly hinders timely diagnosis, especially when patients are in sinus rhythm, as the ECG often appears normal and lacks distinct pathological features. In many cases, invasive electrophysiological studies are required for definitive diagnosis. Moreover, the ECG manifestations during arrhythmic episodes are often similar between AVNRT and CAP, making them difficult to distinguish using conventional visual inspection.

Although both AVNRT and CAP are reentrant tachycardias, they differ in anatomical reentry pathways, leading to different underlying mechanisms and ablation strategies. Consequently, accurate preoperative differentiation is clinically important for tailoring treatment plans, optimizing ablation tools, and minimizing procedural risks. Therefore, developing an automated method capable of identifying AVNRT and CAP from routine sinus rhythm ECGs would be of significant clinical value.

Convolutional neural networks (CNNs) have shown state‐of‐the‐art performance in various medical imaging tasks due to their ability to extract hierarchical spatial features [9, 10, 11, 12]. Their application to ECG analysis has also yielded promising results, enabling the prediction of arrhythmias [13, 14, 15], electrolyte imbalances [16], anemia [17], and sleep apnea [18]. However, the problem of differentiating AVNRT and CAP from sinus rhythm ECGs using deep learning remains unexplored.

To address this gap, we propose CSPANet, a CNN‐based model designed for the prediction of AVNRT, CAP, and normal cases from sinus rhythm ECGs. The model incorporates a Channel and Spatial Parallel Attention (CSPA) module, which processes attention in both channel and spatial dimensions simultaneously. This dual‐path structure enhances the network's ability to capture critical information by adaptively weighting features relevant to specific ECG leads and temporal patterns.

Attention mechanisms, inspired by the human visual and cognitive system, enable selective focus on salient information in complex environments. In deep learning, attention modules have been widely adopted to enhance the model's ability to extract and prioritize meaningful features from high‐dimensional data [19]. These mechanisms also optimize computational efficiency by allocating resources to the most informative components of the input, thereby improving both accuracy and performance [20]. The most common forms of attention mechanisms include channel attention, spatial attention, and their combinations.

Channel attention allows neural networks to learn what to focus on by modeling inter‐channel relationships. Hu et al. proposed the Squeeze‐and‐Excitation Network (SENet), which applies a squeeze‐and‐excitation operation to adaptively recalibrate channel‐wise feature responses, thereby enhancing representational power [21]. To improve efficiency, Wang et al. introduced the Efficient Channel Attention Network (ECANet), which preserves channel dimensions while capturing local cross‐channel dependencies without dimensionality reduction [22]. Similarly, Li et al. developed the Selective Kernel Network (SKNet), which adaptively selects convolutional kernels to adjust receptive fields and improve channel‐wise feature extraction [23].

Spatial attention focuses on *where* to attend by emphasizing important regions in the spatial domain. Hu et al. proposed the Gather‐Excite Network (GENet), which aggregates contextual information over large spatial areas and redistributes it to local features, guiding the model to relevant spatial locations [24]. Jaderberg et al. introduced the Spatial Transformer Network (STNet), which enables the model to perform spatial transformations, such as scaling, translation, and rotation, to dynamically locate informative regions in the input [25].

To further improve feature representation, combined attention mechanisms integrate both channel and spatial attention. For example, Woo et al. proposed the convolutional block attention module (CBAM), which sequentially applies channel and spatial attention to adaptively refine feature maps [26]. Hou et al. introduced coordinate attention (CA), a lightweight module that embeds positional information into channel attention by aggregating features along spatial coordinates, thereby enhancing direction‐ and location‐sensitive representations [27].

In this study, we aim to differentiate AVNRT, CAP, and healthy individuals using sinus rhythm ECG signals. This task involves identifying subtle, latent features that are typically imperceptible to human interpretation. By integrating an attention mechanism specifically designed for ECG data, the model can selectively focus on informative leads or waveform segments, thereby enhancing its ability to learn class‐discriminative patterns and improving overall prediction performance.

## 2 Materials and Methods

**FIGURE 1:**
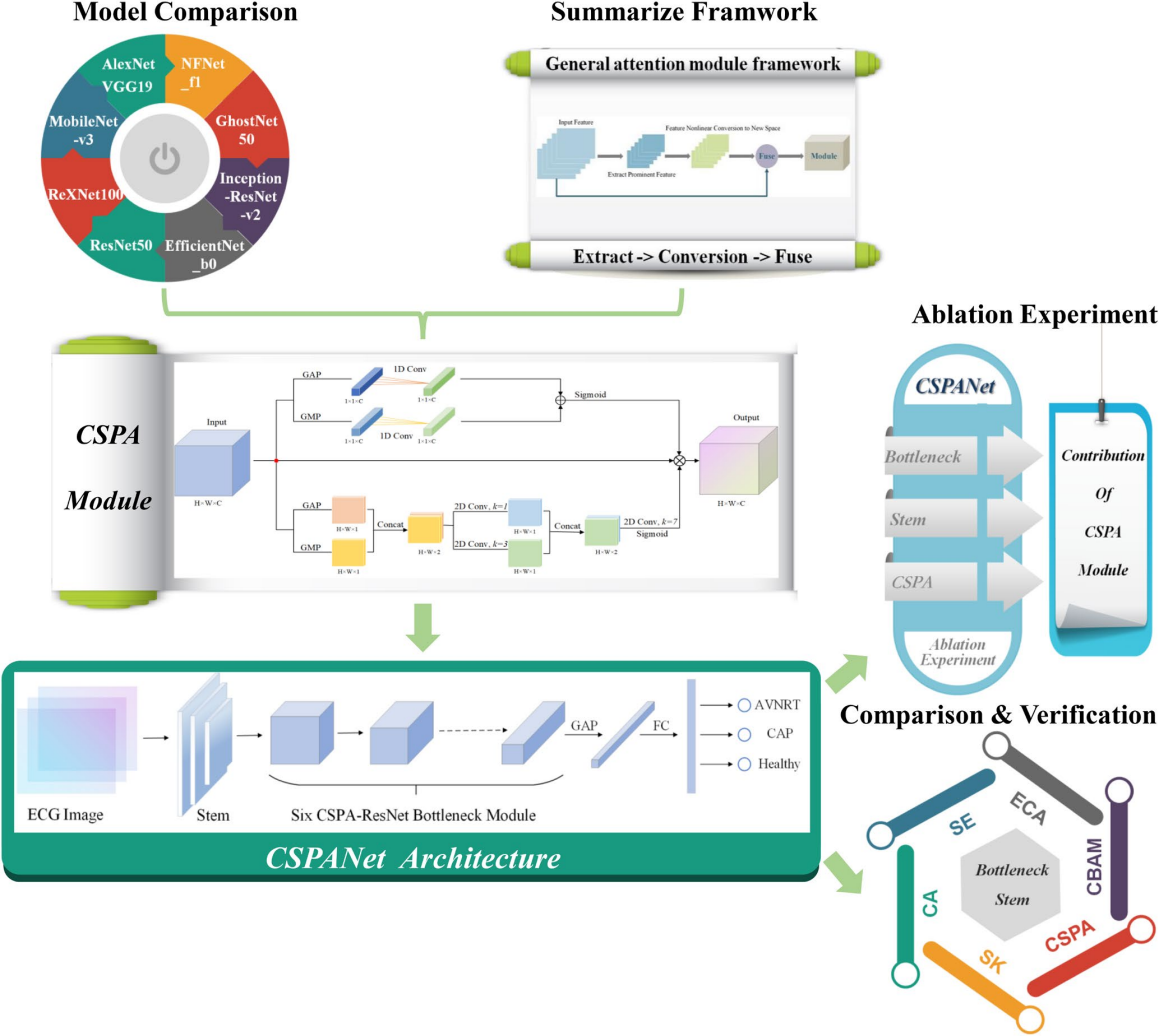
A schematic representation of experimental technical route.

The schematic representation of the experimental technical route is given in Figure 1. The study begins by evaluating the predictive performance of several classical CNN architectures on the target ECG dataset to select an optimal base model as the backbone. Concurrently, existing attention mechanisms are reviewed, and a CSPA module is designed to align with the characteristics of ECG signals, forming the core of the proposed CSPANet model. Subsequently, representative attention modules are integrated into the same backbone for comparison, enabling a fair evaluation of the CSPA module's effectiveness in improving prediction performance. Finally, ablation experiments are conducted to further assess the individual contribution of the channel and spatial attention components within the CSPA module.

### 2.1 General Framework of Attention Modules

**FIGURE 2:**
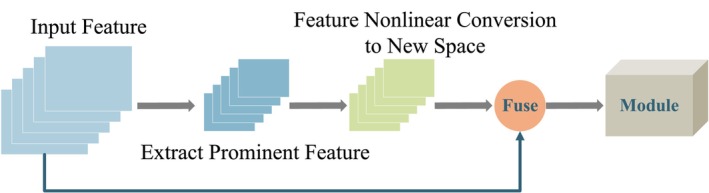
Schematic diagram of the general attention module framework.

In deep learning, attention mechanisms serve as dynamic selection processes that focus on the most relevant information for a given task by adaptively assigning weights to different input features based on their importance [28, 29, 30]. To better understand the design principles of attention mechanisms, various representative modules are analyzed, leading to the formulation of a general attention module framework, illustrated in Figure 2.

Typically, attention modules consist of three sequential components: extraction, transformation, and fusion. The following sections provide a detailed description of each step in the attention mechanism implemented within the general framework.

#### 2.1.1 Extraction

This step selects salient information from the input feature map, denoted as $X\in {\mathbb{R}}^{H\times W\times C}$, where *H*, *W* and *C* represent the height, width, and number of channels, respectively. The extraction step processes the input feature map to produce an output $E=e\left(X,w_{e}\right)$, where *w*_*e*_ represents the parameters involved in the extraction process, and *e* is an extractor.

Most existing attention modules, such as SENet, ECANet, and CANet, utilize global average pooling or global max pooling as the primary extraction strategy to capture global contextual information.

#### 2.1.2 Transformation

The extracted prominent features are projected into a new attention space through a nonlinear transformation. Let *t* represent the transformer and *w*_*t*_ denote the parameters involved in the transformation process. The output features after transformation are $T=t\left(E,w_{t}\right)$. Non‐linear transformations are typically implemented using fully connected layers, activation functions, 1 × 1 convolutional layers, or a combination of these.

#### 2.1.3 Fusion

The attention map obtained from the transformation step is combined with the input feature map. The final attention output features are defined as $\bar{X}\in {\mathbb{R}}^{H\times W\times C}$, expressed by the formula $\bar{X}=f\left(X,T\right)$, where *f* represents the fusion operation. The fusion can be performed through element‐wise multiplication or addition.

In summary, the modeling equation for the attention module can be expressed as follows:

(1) $$\bar{X}=f\left(t\left(e\left(X,w_{e}\right),w_{t}\right),X\right)=t\left(e\left(X,w_{e}\right),w_{t}\right)⊙X$$

In the equation, the symbol ⊙can represent either the addition operation ⊕ or the multiplication operation ⊗. By combining the attention map generated by the attention mechanism with the original input feature map, the model effectively assigns attention weights to the original input features.

### 2.2 Channel and Spatial Parallel Attention Module

**FIGURE 3:**
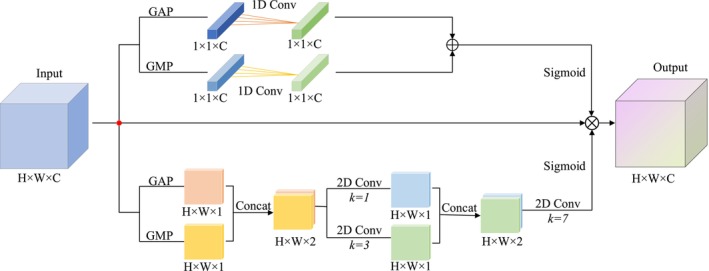
Architecture of the proposed CSPA module.

The proposed CSPA module comprises two submodules: channel attention and spatial attention. It adopts a parallel attention architecture, enabling simultaneous processing of attention across both channel and spatial dimensions. This parallel strategy improves the efficiency and effectiveness of feature refinement, thereby enhancing overall model performance. The structural overview of the CSPA module is shown in Figure 3.

#### 2.2.1 Channel Attention Submodule

**FIGURE 4:**
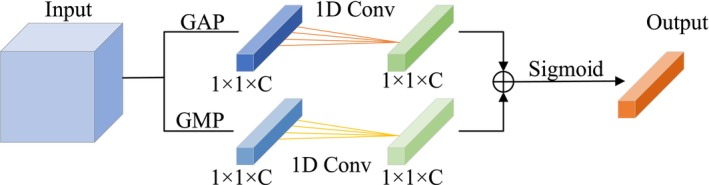
Architecture of the proposed channel attention submodule.

The channel attention mechanism models attention weights for each feature channel, quantifying their relative importance. Given the complex and subtle nature of ECG signal features across different frequency bands, we adopt a dual‐path strategy to compute two distinct channel attention factors. These are subsequently fused to enable the model to assign higher weights to more informative channels, thereby reinforcing critical information in the input feature map. The structure of the proposed channel attention submodule is depicted in Figure 4.

The input feature map is first processed using both Global Average Pooling (GAP) and Global Max Pooling (GMP) operations. These operations compress the spatial dimensions (height and width), producing two distinct channel‐wise descriptors of size 1 × 1 × C, where C is the number of channels. Inspired by the ECA module [22], we avoid dimensionality reduction and apply 1D convolution directly to the pooled vectors. Specifically, a 1D convolution with kernel size *k* is used to enable local cross‐channel interaction, allowing each channel to interact with its 𝑘‐nearest neighbors. The resulting channel attention map is computed as follows:

(2) $$\begin{array}{c} W^{c}=\sigma \left(C1D\left(f_{\max}^{c}\left(X\right)\right)+C1D\left(f_{\mathrm{avg}}^{c}\left(X\right)\right)\right)\\ \;=\sigma \left(C1D\left(y_{\max}^{c}\right)+C1D\left(y_{\mathrm{avg}}^{c}\right)\right) \end{array}$$

In the equation, given the input feature map $X\in {\mathbb{R}}^{H\times W\times C}$, *W* ^*c*^ is the attention factor assigned to each channel. $f_{\max}^{c}$ represents the Global Max Pooling operation, $f_{\mathrm{avg}}^{c}$represents the global average pooling operation. $y_{\max}^{c}$ represents the channel feature obtained after the max pooling operation, $y_{\max}^{c}\in {\mathbb{R}}^{1\times 1\times C}$. $y_{\mathrm{avg}}^{c}$ is the channel feature obtained after the average pooling operation, $y_{\mathrm{avg}}^{c}\in {\mathbb{R}}^{1\times 1\times C}$. *σ* is the Sigmoid function. C1D refers to the 1D convolution, which facilitates cross‐channel interactions between channels and their neighbors.

This approach allows the model to selectively enhance informative channels based on both average and maximum feature activations, improving its capacity to capture subtle yet important ECG signal characteristics.

#### 2.2.2 Spatial Attention Submodule

The spatial attention mechanism operates on the spatial dimensions of the input feature map, transforming spatial information through a convolutional module to generate location‐specific attention weights. This process preserves essential spatial cues and enhances the representation of locally important features. By assigning distinct weights to different spatial positions, the model is guided to focus on regions that are more informative for the task.

**FIGURE 5:**
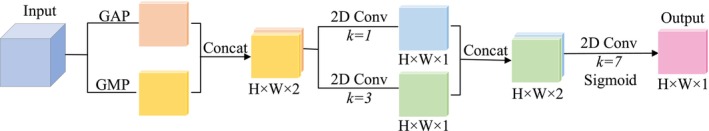
Architecture of the proposed spatial attention submodule.

In the context of ECG‐based disease diagnosis, spatial attention is particularly relevant, as the diagnostic process often relies on analyzing waveform morphology across different frequency bands and evaluating the temporal distances between them. These features are reflected as positional patterns on the ECG, making spatial information critical for accurate interpretation. Therefore, enhancing the model's sensitivity to effective spatial regions is essential. To address this need, we design a spatial attention submodule, as illustrated in Figure 5.

First, for each feature point in the input feature map, GAP and GMP operations are applied along the channel direction to obtain the average pooled and max pooled characteristics of the channels, generating two 2D feature maps. Next, these two 2D feature maps are stacked together and then passed through two parallel 2D convolution operations. Without changing the size of the feature maps, 2D convolutions with kernel sizes of 1 and 3 are applied to generate richer feature maps. Then, the feature maps obtained from the convolution operations are stacked again to create a two‐channel 2D feature map. A standard convolution layer (with a channel size of 1) is used for concatenation and convolution. Finally, a Sigmoid function is used to obtain the attention factor for each feature point in the input feature map (ranging from 0 to 1), and the resulting spatial attention factor is multiplied and weighted to each feature point in the input feature map. The spatial attention factor is calculated using the formula (3).

(3) $$W^{s}=\sigma \left(C^{7\times 7}\left[C^{1\times 1}\left[f_{\max}^{s}\left(X\right);f_{\mathrm{avg}}^{s}\left(X\right)\right];C^{3\times 3}\left[f_{\max}^{s}\left(X\right);f_{\mathrm{avg}}^{s}\left(X\right)\right]\right]\right)$$

In the equation, given the input feature map $X\in {\mathbb{R}}^{H\times W\times C}$, *W* ^*s*^ is the attention factor assigned to each spatial location. $f_{\max}^{s}$ represents the Global Max Pooling operation along the channel direction. $f_{\mathrm{avg}}^{s}$ represents the Global Average Pooling operation along the channel direction. [;] refers to the operation of stacking the two feature maps before and after the pooling operations. C^1 × 1^ represents the 1 × 1 × C convolution kernel. C^3 × 3^ represents the 3 × 3 convolution kernel. C^7 × 7^ represents the 7 × 7 convolution kernel. *σ* is the Sigmoid function.

Finally, the initial input feature map is multiplied by the attention factors from each dimension, resulting in the attention‐weighted output of the CSPA module.

(4) $$\text{Output}=\text{Input}*W^{c}*W^{s}$$

In this way, the CSPA module simultaneously captures critical information from both the channel and spatial dimensions.

### 2.3 Overall Framework Design of the Prediction Model

**FIGURE 6:**
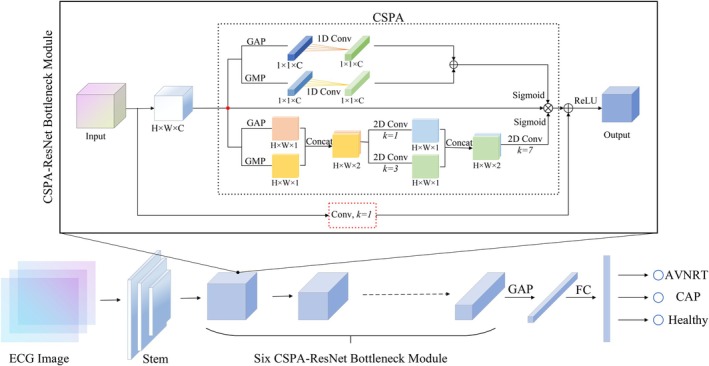
Overall architecture of the proposed CSPANet model.

The proposed CSPANet model primarily comprises a Stem module and six CSPA‐ResNet bottleneck modules. The Stem module adopts an inverted triangular structure, consisting of three sequential convolutional layers with kernel sizes of 7 × 7, 5 × 5 and 3 × 3, respectively. This design aims to extract as many edge features of the ECG waveforms as possible. To enhance the model's ability to focus on critical and subtle ECG characteristics, the CSPA module is integrated into the residual bottleneck block, forming the CSPA‐ResNet bottleneck module. The overall architecture of CSPANet is depicted in Figure 6. An example from one of the six CSPA‐ResNet bottleneck modules is provided. The black dashed box contains the designed CSPA attention mechanism. The red dashed box contains the 1 × 1 convolution, primarily used for matching the number of channels. When the input and output channel dimensions of the module are the same, an identity shortcut connection is used, and the 1 × 1 convolution is omitted. When the input and output channel dimensions differ, a projection shortcut connection is employed, which requires a 1 × 1 convolution to achieve compatibility.

### 2.4 Experimental Environment and Hyperparameter Settings

All experiments were conducted using the PyTorch deep learning framework, with additional support from libraries such as NumPy and Matplotlib. The experimental environment was based on Linux CentOS 7.8 and Python 3.6. Model training was accelerated using an NVIDIA A100 PCIe GPU with 40 GB of memory. All experiments were performed on a cloud computing platform. The hyperparameter settings for training the CSPANet model are summarized in Table 1.

**TABLE 1:** Hyperparameter settings for the CSPANet model.

Hyper parameter	Symbol	Set VALUE
Input image resolution	(W, H)	(1600, 800)
Batch size	BS	8
Initial learning rate	Α	0.0001
Learning rate strategy	LR	Cosine Annealing

In this experiment, due to the large parameter size of the NFNet_f1 model and GPU memory limitations, the input ECG resolution for this model was adjusted to 1024 × 512. During training, the model was optimized using the Adam optimizer and the cross‐entropy loss function over 100 epochs. The cross‐entropy loss function is defined as follows:

(5) $$\text{loss}=-\left(y_{0}\log\left(a_{0}\right)+y_{1}\log\left(a_{1}\right)+y_{2}\log\left(a_{2}\right)\right)$$

where *y*_0_, *y*_1_, *y*_2_ represent the ground truth labels for classes 0, 1, and 2, respectively, while *a*_0_, *a*_1_, *a*_2_ represent the predicted probabilities for classes 0, 1, and 2, respectively.

### 2.5 Dataset Division for Three‐Class Classification

The dataset used in this study comprises standard 10‐s, 12‐lead ECG recordings, including leads I, II, III, V1‐V6, aVR, aVL, and aVF. These ECGs were acquired using MAC 800 or 1200ST electrocardiograph machines (GE Healthcare) at a sampling frequency of 500 Hz. A total of 1143 ECG recordings were collected from patients who underwent electrophysiological examinations and radiofrequency ablation procedures between January 1, 2013, and August 31, 2021. Among these, 443 patients were classified into the CAP group and 700 into the AVNRT group. Additionally, the control group consisted of ECGs from 5107 individuals who visited Wuxi People's Hospital for routine ECG examinations between January 1 and March 10, 2020. This study was approved by the Ethics Committee of the Affiliated Wuxi People's Hospital of Nanjing Medical University. All work was conducted in accordance with the Declaration of Helsinki (1964).

ECG images output from ECG processing system were used in this study to develop a tool that physicians could be capable of directly analyzing ECG reports stored in the image format most commonly encountered in daily practice. The collected ECG images were categorized into three groups: the CAP group, the AVNRT group, and a healthy control group without any documented symptoms, signs, or clinical history of AVNRT or CAP. The inclusion criteria for the AVNRT and CAP groups were as follows: (1) Patients with a clinical diagnosis of AVNRT or CAP, based on the presence of typical paroxysmal palpitations characterized by sudden onset and termination. (2) Diagnosis confirmed by electrophysiological study and successful radiofrequency catheter ablation. (3) Availability of a standard sinus rhythm ECG recorded prior to the electrophysiological examination.

**FIGURE 7:**
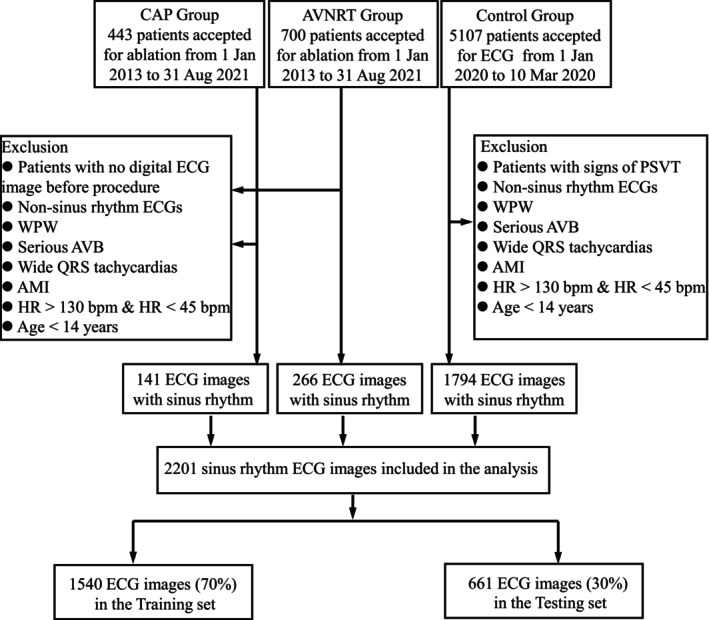
Workflow for dataset construction.

The control group comprised individuals evaluated by cardiologists through outpatient consultations, review of medical records, or telephone follow‐up, and confirmed to have no evidence or history of AVNRT or CAP. ECGs from all groups were screened using consistent exclusion criteria, including the removal of cases involving individuals under 14 years of age. Additionally, none of the participants in any group were receiving antiarrhythmic medications at the time of ECG acquisition. The workflow for dataset construction is illustrated in Figure 7.

The three filtered ECG categories were randomly divided into training and testing sets at a 7:3 ratio, with no patient overlap between the two subsets (i.e., ECGs from the same patient appear in only one dataset). Prior to model training, raw ECG recordings were cropped to retain only the relevant waveform segments, thereby eliminating extraneous metadata. Subsequently, bilinear interpolation was applied to uniformly rescale the cropped signals, standardizing input resolution across all samples.

### 2.6 Evaluation Metrics

In the experiments, ECG data from healthy individuals were labeled as class 0, ECG data from CAP patients as class 1, and ECG data from AVNRT patients as class 2. The performance of the models was evaluated using various metrics, including accuracy, precision, sensitivity, and specificity.

(6) $$\text{Accuracy}=\frac{\left({\mathrm{TP}}_{0}+{\mathrm{TP}}_{1}+{\mathrm{TP}}_{2}\right)}{\left({\mathrm{TP}}_{0}+{\mathrm{FN}}_{0}+{\mathrm{TP}}_{1}+{\mathrm{FN}}_{1}+{\mathrm{TP}}_{2}+{\mathrm{FN}}_{2}\right)}$$

(7) $$\text{Sensitivity}=\left(\frac{{\mathrm{TP}}_{0}}{{\mathrm{TP}}_{0}+{\mathrm{FN}}_{0}}+\frac{{\mathrm{TP}}_{1}}{{\mathrm{TP}}_{1}+{\mathrm{FN}}_{1}}+\frac{{\mathrm{TP}}_{2}}{{\mathrm{TP}}_{2}+{\mathrm{FN}}_{2}}\right)\times \frac{1}{3}$$

(8) $$\text{Specificity}=\left(\frac{{\mathrm{TN}}_{0}}{{\mathrm{TN}}_{0}+{\mathrm{FP}}_{0}}+\frac{{\mathrm{TN}}_{1}}{{\mathrm{TN}}_{1}+{\mathrm{FP}}_{1}}+\frac{{\mathrm{TN}}_{2}}{{\mathrm{TN}}_{2}+{\mathrm{FP}}_{2}}\right)\times \frac{1}{3}$$

(9) $$\text{Precision}=\left(\frac{{\mathrm{TP}}_{0}}{{\mathrm{TP}}_{0}+{\mathrm{FP}}_{0}}+\frac{{\mathrm{TP}}_{1}}{{\mathrm{TP}}_{1}+{\mathrm{FP}}_{1}}+\frac{{\mathrm{TP}}_{2}}{{\mathrm{TP}}_{2}+{\mathrm{FP}}_{2}}\right)\times \frac{1}{3}$$

where *TP*_*i*_ represents the number of individuals correctly predicted as belonging to class *i*. *FP*_*i*_ represents the number of individuals incorrectly predicted as belonging to class *i*. *FN*_*i*_ represents the number of individuals belonging to class *i* but predicted as not belonging to class *i*. *TN*_*i*_ represents the number of individuals not belonging to class *i* and correctly predicted as not belonging to class *i*. Here, *i* takes the values 0, 1, and 2.

## 3 Results

### 3.1 Patient Baseline Clinical Characteristics

The patient baseline clinical characteristics of these three groups were summarized in Table 2. The mean age of patients was 47.2 ± 16.5 years in the control group, 49.7 ± 14.6 years in the CAP group, and 54.3 ± 14.9 years in the AVNRT group. There were no statistically significant differences among the control group, CAP group, and AVNRT group in terms of gender, P‐R interval, QRS interval, and QT interval. Statistically significant differences were observed in age, heart rate, and QTc interval.

**TABLE 2:** Patient baseline clinical characteristics.

Parameters	Control group (*n* = 1794)	CAP group (*n* = 141)	AVNRT GROUP (*n* = 266)	Total (*n* = 2201)	*p*
Age (years)	47.2 ± 16.5	49.7 ± 14.6	54.3 ± 14.9	48.2 ± 16.4	< 0.001
Gender (Male, %)	835 (46.5)	68 (48.2)	115 (43.2)	1018 (46.3)	0.533
HR (bpm)	80.6 ± 14.7	78.2 ± 14.2	77.8 ± 13.6	80.1 ± 14.6	0.004
P‐R interval (ms)	149.5 ± 19.1	150.0 ± 19.4	151.5 ± 23.7	149.8 ± 19.7	0.330
QRS interval (ms)	84.7 ± 11.7	85.0 ± 10.5	85.2 ± 12.3	84.8 ± 11.7	0.753
QT interval (ms)	363.6 ± 32.0	356.9 ± 30.1	363.1 ± 32.2	363.1 ± 32.2	0.058
QTc (ms)	416.7 ± 25.9	406.8 ± 25.8	413.7 ± 26.8	415.8 ± 26.1	< 0.001

### 3.2 The Performance of Classical Models

To develop an effective model for distinguishing healthy individuals, CAP patients, and AVNRT patients based on sinus rhythm electrocardiograms, nine classical CNN architectures were trained and evaluated. Model performance was assessed using accuracy, sensitivity, specificity, precision, and confusion matrices, as summarized in Table 3.

**TABLE 3:** Evaluation metrics of different models on the test set.

Models	Accuracy	Sensitivity	Specificity	Precision	Confusion matrix
AlexNet	0.867	0.628	0.881	0.651	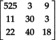
VGG19	0.852	0.601	0.884	0.595	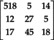
NFNet_f1	*0.890*	0.622	0.902	0.663	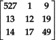
GhostNet50	0.825	0.564	0.879	0.599	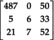
Inception‐ResNet‐v2	**0.897**	0.681	0.924	**0.695**	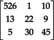
EfficientNet_b0	0.885	0.640	0.901	0.693	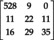
ReXNet100	0.877	0.630	0.883	0.676	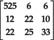
ResNet50	0.858	**0.695**	**0.927**	0.621	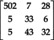
MobileNet‐v3	0.868	0.605	0.852	0.681	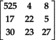

*Note:* Bold values indicate the best performance for each evaluation metric.

Among the tested models, ResNet50 and Inception‐ResNet‐v2 demonstrated superior performance across multiple metrics. Both networks leverage residual learning, highlighting its effectiveness in enhancing model accuracy. Notably, ResNet50 achieved the highest sensitivity and specificity, two critical metrics in clinical classification tasks, thereby validating the use of the bottleneck residual module as a robust structural foundation for ECG‐based predictive modeling.

### 3.3 Comparison of Different Attention Mechanisms

In the comparative experiments, the ResNet22 model‐comprising a stem module, six bottleneck residual modules, and an output layer‐was adopted as the backbone network. The proposed CSPA mechanism was benchmarked against several representative attention mechanisms introduced in recent years, including SE [21], ECA [22], CBAM [26], CA [27], and SK [23]. The experimental results are summarized in Table 4.

**TABLE 4:** Evaluation metrics of models with different attention mechanisms on the test set.

Backbone	Attention mechanism	Accuracy	Sensitivity	Specificity	Precision	Confusion matrix
ResNet22	+ SE	*0.897*	0.706	*0.932*	0.701	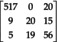
	+ ECA	0.877	*0.742*	0.927	0.675	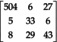
	+ CBAM	0.891	0.667	0.918	0.697	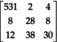
	+ CA	0.896	0.680	0.907	*0.724*	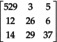
	+ SK	0.896	0.677	0.915	0.697	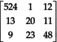
	+ CSPA	**0.926**	**0.790**	**0.950**	**0.797**	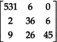

*Note:* Bold values indicate the best performance for each evaluation metric, while italicized values denote the second‐best results.

As shown in Table 4, the model incorporating the CSPA module in the backbone network outperforms those equipped with other representative attention mechanisms across all evaluation metrics on the test set. Specifically, it achieved an accuracy of 0.926, sensitivity of 0.790, specificity of 0.950, and precision of 0.797. Compared to the second‐best performance, the inclusion of the CSPA module led to a 4.8% improvement in sensitivity, a 2.9% increase in accuracy, a 1.8% gain in specificity, and a 7.3% enhancement in precision. These results demonstrate that the proposed CSPA mechanism surpasses the five representative attention mechanisms in boosting model performance. By effectively guiding the model to focus on salient spatial and channel‐wise information in the input feature maps, the CSPA mechanism enhances the model's capacity to extract relevant ECG features. Consequently, it significantly improves the model's ability to distinguish and predict CAP and AVNRT patients from sinus rhythm ECGs.

### 3.4 Ablation Experiment

The CSPANet model primarily consists of the Stem module and the CSPA‐ResNet Bottleneck module. It is built upon the ResNet20 architecture, which includes only bottleneck residual modules. The analysis focuses on the performance improvements achieved by incorporating the Stem and CSPA modules into CSPANet. The results of the ablation experiments are presented in Table 5. Notably, replacing the Stem module in ResNet22 with a single convolutional layer with a kernel size of 7 yields the ResNet20 configuration.

**TABLE 5:** Results of ablation experiments.

Stem	CSPA	Accuracy	Sensitivity	Specificity	Precision	Confusion matrix
		0.897	0.673	0.918	0.709	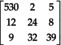
**√**		0.896	0.638	0.896	0.719	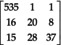
	√	*0.915*	*0.720*	*0.947*	*0.720*	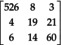
**√**	√	**0.926**	**0.790**	**0.950**	**0.797**	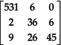

*Note:* Bold values indicate the best performance for each evaluation metric, while italicized values denote the second‐best results.

As shown in Table 5, retaining only the bottleneck residual modules and introducing the Stem module alone improves the model's accuracy by 1%. Incorporating the CSPA module independently further enhances model performance, increasing accuracy by 1.8%, sensitivity by 4.7%, specificity by 2.9%, and precision by 1.1%, achieving the second‐best results across all these metrics. When both the Stem and CSPA modules are integrated into the CSPANet model, it achieves optimal performance across all evaluation metrics on the test set. Compared with the ResNet20 model, the accuracy increases by 2.9%, sensitivity by 11.7%, specificity by 3.2%, and precision by 8.8%.

**FIGURE 8:**
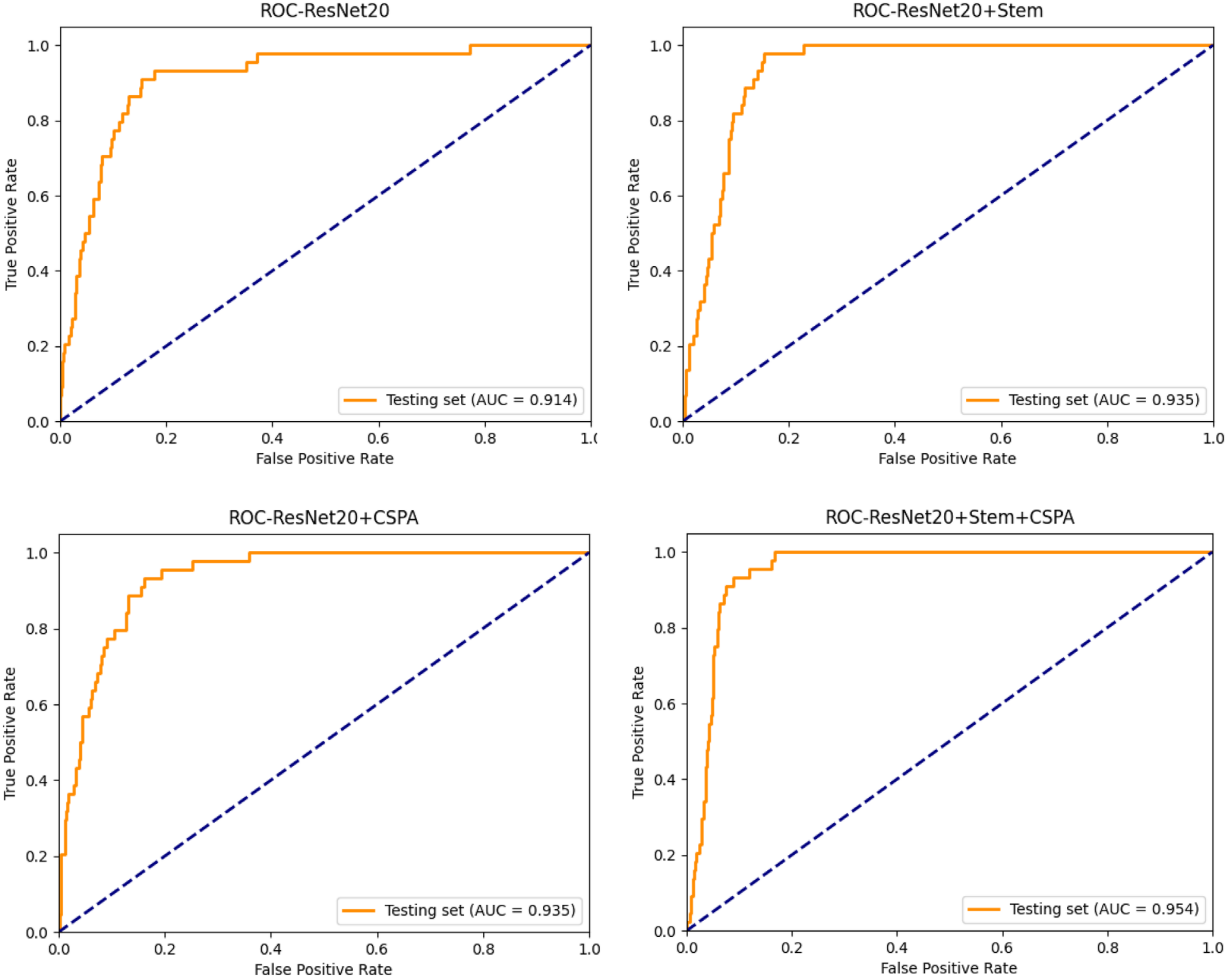
ROC curve and AUC.

To further illustrate the performance improvements contributed by each module, the ROC curve was plotted and the area under the curve (AUC) was calculated, as shown in Figure 8.

A comprehensive evaluation of the metrics in Table 5 and the AUC values of each model on the test set in Figure 8 indicates that both the CSPA module and the Stem module enhance the model's predictive performance. However, the CSPA module demonstrates a more substantial impact. When both the CSPA and Stem modules are integrated, the model achieves its highest overall performance.

## 4 Discussion

This study introduces CSPANet, a novel deep learning architecture equipped with a Channel and Spatial Parallel Attention (CSPA) module, for the non‐invasive differentiation of CAP and AVNRT from standard sinus rhythm ECGs. Our model demonstrated superior performance, achieving high accuracy, sensitivity, and specificity, significantly outperforming both classical CNN architectures and existing attention mechanisms. The following points discuss the implications, strengths, and limitations of our findings.

### 4.1 Addressing a Critical Clinical Diagnostic Gap

The primary significance of this work lies in its direct address of a persistent challenge in clinical electrophysiology: the inability to preoperatively distinguish between CAP and AVNRT using conventional sinus rhythm ECG analysis. This diagnostic ambiguity often forces reliance on invasive electrophysiological studies for definitive diagnosis, which carry inherent risks and resource burdens. CSPANet's ability to identify subtle, latent electrophysiological substrates from a routine, non‐invasive ECG presents a paradigm shift. By providing a probable diagnosis before an invasive procedure, this tool can guide clinical decision‐making, allowing electrophysiologists to better plan ablation strategies, select appropriate equipment, and counsel patients on specific procedural risks and success rates, ultimately optimizing the entire pathway of care.

### 4.2 Generalizability and Clinical Translation Potential

The robust performance of CSPANet across a diverse dataset is promising for its generalizability. The model was trained and tested on ECGs acquired from real‐world clinical settings using common GE Healthcare electrocardiographs, enhancing its potential for practical deployment. Furthermore, by cropping and standardizing the raw ECG signals, we developed a method that is less sensitive to variations in ECG printout formats or metadata across different hospital systems. This preprocessing step, combined with the model's strong performance, suggests that CSPANet could be integrated into existing hospital digital ECG management systems as a decision‐support tool, flagging potential underlying substrates during routine sinus rhythm ECG interpretation.

### 4.3 Limitations and Future Directions

Despite its promising results, this study has several limitations. First, the data was sourced from a single center, which may introduce selection bias. External validation on multi‐center, international datasets is crucial to confirm the model's robustness and generalizability across diverse populations and ECG machine vendors. Second, the model is currently a “black box” in terms of precise feature identification. While the attention maps provide insight into areas the model focuses on, a more detailed explainability analysis, such as using Gradient‐weighted Class Activation Mapping (Grad‐CAM) to highlight specific ECG waveforms, would strengthen clinical trust and adoption. Third, the use of ECG images rather than raw signals may entail information loss and susceptibility to non‐clinical artifacts; although access to raw digital signals could potentially enhance model performance, our approach demonstrates the feasibility of leveraging the vast repository of unstructured ECG image data commonly encountered in real‐world clinical practice. Fourth, although the three groups were well balanced for several clinical parameters, statistically significant differences were observed in age, heart rate, and QTc interval. While these differences reflect the inherent clinical heterogeneity of the populations (e.g., patients with AVNRT are typically older), they may act as potential confounders. Future work using larger, matched cohorts or multivariable adjustment to strengthen causal inference, as well as a focus on explainability and prospective validation in a live clinical environment, will be essential to assess real‐world impact on diagnostic yield and patient outcomes.

## 5 Conclusion

This study highlights the potential of deep learning to improve the accuracy and efficiency of cardiovascular disease diagnosis, especially for complex cases such as AVNRT and CAP using non‐invasive ECG images. The proposed CSPANet model, equipped with a novel attention mechanism, presents a promising tool for aiding clinical decision‐making and facilitating early intervention, ultimately contributing to more personalized and effective patient care.

## Funding

This study was supported by grants from Top Talent Support Program for young and middle‐aged people of Wuxi Health Committee (grant number: HB2023006) and 2025 Jiangsu Province “Qing Lan Project” Outstanding Teaching Team (Intelligent Equipment Control Technology Teaching Team).

## Ethics Statement

This study was approved by the Ethics Committee of the Affiliated Wuxi People's Hospital of Nanjing Medical University. All work was conducted in accordance with the Declaration of Helsinki (1964).

## Conflicts of Interest

The authors declare no conflicts of interest.

## Data Availability

Data are available on request.
